# Associations Between Phenotypes of Childhood and Adolescent Obesity and Incident Hypertension in Young Adulthood

**DOI:** 10.21203/rs.3.rs-4113605/v1

**Published:** 2024-03-21

**Authors:** Ruth St Fleur, Marian Tanofsky-Kraff, Jack Yanovski, Nicholas Horton, Laura Reich, Jorge chavarro, Joel Hirschhorn, Hannah Ziobrowski, Alison Field

**Affiliations:** Brown University; Uniformed Services University (USU); National Institutes of Health; Amherst College; Brown University School of Public Health; Boston Children’s Hospital; Brown University School of Public Health; Brown University School of Public Health

## Abstract

**Objectives:**

We investigated whether empirically derived childhood obesity phenotypes were differentially associated with risk of hypertension in young adulthood, and whether these associations differed by sex.

**Methods:**

Data came from 11,404 participants in the Growing Up Today Study, a prospective cohort study in the US established in 1996. We used a childhood obesity phenotype variable that was previously empirically derived using latent class analysis. The childhood obesity phenotypes included an early puberty phenotype (females only), a mothers with obesity phenotype, a high weight concerns phenotype, and a mixed phenotype. Participants without overweight or obesity in childhood or adolescence were the reference group. We then used logistic regression models with generalized estimating equations to examine associations of childhood obesity phenotypes with incident hypertension between ages 20–35 years. All analyses were stratified by sex.

**Results:**

Among females, participants in all of the empirically derived childhood obesity phenotypes were more likely than their peers without childhood overweight/obesity to develop hypertension in young adulthood (early puberty subtype odds ratio (OR) = 2.52; 95% confidence interval (CI) = 1.75, 3.62; mothers with obesity (MO) subtype OR = 2.98; 95% CI = 1.93, 4.59; high weight concerns (WC) subtype OR = 2.33; 95% CI = 1.65, 3.28; mixed subtype OR = 1.66; 95% CI = 1.25, 2.20). Among males, the childhood obesity phenotypes were associated with a higher risk of developing hypertension, although males in the MO (OR = 2.65; 95% CI = 1.82, 3.87) and WC phenotypes (OR = 3.52; 95% CI = 2.38, 5.20) had a greater risk of developing hypertension than the mixed subtype (OR = 1.51; 95% CI = 1.23, 1.86) (*p* = 0.004).

**Conclusion:**

Risk for incident hypertension in young adulthood varied by childhood obesity phenotypes, as well as by biological sex. If replicated, these results may suggest that increased surveillance of specific childhood obesity phenotypes might help in targeting those at highest risk for hypertension.

## Introduction

Individuals with hypertension are at increased risk for heart disease, stroke, chronic kidney disease, other cardiovascular diseases (CVDs) and premature mortality.^1–3^ Moreover, hypertension is a major contributor to healthcare expenditure among adults in the US.^4^ Although traditionally more prevalent in older populations, hypertension has been increasing in incidence in younger adults in the US.^5, 6^ Recent estimates suggest that approximately 22% of young adults in the US have hypertension.^7^ There is evidence that hypertension is established early in life,^8^ rendering childhood and adolescence critical periods for prevention. Obesity and elevated blood pressure in childhood are among the more studied risk factors for hypertension in young adulthood.^9–11^ Although adult obesity is a critical risk factor for adult hypertension, evidence from a pooled cohort study suggests that having obesity during both childhood and adulthood may confer a greater risk of adult hypertension than having obesity only as an adult, highlighting the long lasting impact of childhood risk factors on health across the life course.^12^

Although childhood weight status is a robust predictor for later developing components of the metabolic syndrome, including hypertension, not all individuals with obesity develop adverse cardiometabolic health consequences. Such individuals have been referred to as having metabolically healthy obesity (MHO). It is likely that biological and behavioral factors beyond just weight contribute to metabolic risk for those with obesity, and that the combination of these factors may contribute to the observed differential risks for cardiometabolic health outcomes. As such, it is important to consider these other factors in addition to weight status alone when examining associations of obesity with hypertension risk. Such factors may include pubertal development, disordered eating behaviors, and depression. Pubertal development is accompanied by changes in body composition and hormone levels, which alter risk of hypertension.^13–15^ In addition, disordered eating behaviors (e.g., binge eating) and depressive symptoms often develop during adolescence and may influence CVD risk.^16–19^ Females with earlier age at menarche are more likely to develop both high depressive symptoms and disordered eating behaviors, all of which are more common among girls with overweight or obesity.^20–23^ The association between disordered eating behaviors, pubertal timing, and hypertension has been less studied in males. We have yet to understand how multiple childhood risk factors among people with obesity combine into distinct phenotypes to confer risk for cardiometabolic dysfunction in young adulthood.

To better understand the heterogeneity in the association between weight status and the risk of developing metabolic abnormalities such as hypertension, we leveraged empirically derived obesity phenotypes in childhood and adolescence as predictors of hypertension in young adulthood. Using latent class analysis (LCA), we previously identified distinct childhood and adolescent obesity phenotypes based on psychological, behavioral, and familial risk factors in females and males in the Growing Up Today Study (GUTS).^24^ In prospective analyses, we found that the identified obesity phenotypes were differentially associated with weight gain in young adulthood, indicating their predictive power for cardiometabolic risks and the possibility they might inform tailored intervention strategies. The present study aimed to 1) assess prospective association between empirically derived childhood and adolescent obesity phenotypes and the risk of developing hypertension in young adulthood, and 2) examine difference in these associations by biological sex.

## Methods

### Sample

We used data from GUTS, an ongoing prospective cohort study in the US established in 1996. Participants are children of women in the Nurses’ Health Study II^25^ who had children aged 9–14 years in 1996. These women were asked for consent to invite their children to participate in GUTS, and a total of 9,033 female and 7,843 male children whose mothers consented agreed to be in the study. GUTS questionnaires were sent to participants annually from 1996 to 2001, and then every 2–3 years (with the most recent wave of data collection used in analyses from 2016). As previously described,^24^ LCA was used to develop distinct phenotypes among the 2,655 females and 2,816 males who did not have hypertension but were classified as having overweight or obesity based on their self-reported weight and height, in one or more years between ages 9 and 19 years. The sample for analyses predicting the development of hypertension included these 5,471 youth who did not have hypertension before age 20, as well as 11,404 youth who did not have obesity, and who also provided information on weight and height; all participants also had at least one follow-up after age 19 years. The final sample size was 8,591 females and 7,842 males.

### Measures

#### Obesity Phenotypes:

The obesity phenotypes previously identified through LCA included indicators for disordered eating behaviors, body image and weight concerns, depressive symptoms, pubertal timing, and participants’ and maternal current and childhood weight status. As previously reported,^26^ four phenotypes were observed among females with overweight or obesity between ages 9–19 years: 1) “*Early Puberty*”, defined by having a high probability of early puberty ; 2) “*Mothers with Obesity*”, characterized by high probabilities of maternal history of obesity ; 3) “*High Weight Concerns*”, defined by high probabilities of concerns with body weight and shape, social influence on eating and depression; and 4) “*Mixed*”, defined by low probabilities of all obesity-related indicators (Table 1a). Among males with overweight or obesity between ages 9–19 years, the same phenotypes were observed except for “Early Puberty” since age at menarche was not included as an indicator for males (Table 1b).

#### Outcome:

The outcome was the development of hypertension between ages 20 and 35. In 2010, 2013, 2014, 2015, and 2016, surveys assessed a range of chronic health conditions, including hypertension. Participants were asked, “*Have you ever been told by a healthcare provider that you have any of the following illnesses?*” Those who answered “yes” were asked to indicate the year of the first diagnosis. Participants who indicated they had been diagnosed with hypertension after age 19 were classified as having the outcome of interest.

#### Covariates:

Physical activity was assessed in 1996, 1997, 1998, 1999, and 2001 with the Youth Adolescent Activity Questionnaire.^27^ Participants were asked to report their average hours per week engaged in a variety of activities over the past year. The 1997–2001 questionnaires asked for an average per season (i.e., Fall, Winter, Spring, and Summer). Information from each season was then summed to provide an annual estimate. Vigorous activity was computed as the average hours per week engaged in the following sports: basketball, dance/aerobics, hockey/lacrosse, running, swimming, skating, soccer, tennis, football (males only), cheerleading/gymnastics, volleyball, and martial arts. Participants in the top quartile of vigorous activity were classified as highly active.

Diet was assessed using the Youth/Adolescent Questionnaire, a validated food frequency questionnaire,^28^ in 1996, 1997, 1998, and 2001. This questionnaire included approximately 150 food items and was modified from the validated adult Willett food frequency questionnaire to reflect the cognitive level and dietary knowledge of adolescents.^29^ The questionnaire asked, on average, how often participants had consumed specific foods during the past year and included snack foods consumed by a younger population and food eaten away from home.^29^ We used the Alternate Healthy Eating Index 2010^30^ as a composite measure of diet quality. This diet score summarizes information on intake of vegetables, fruits, nuts, whole grains, polyunsaturated fatty acids, and long chain omega 3 fatty acids as well as lower intake of red and processed meats, sugar sweetened beverages, trans fats, and sodium.

We calculated BMI using self-reported weight and height information when participants were 18 years old. Previous research has shown that these self-reported measures are valid in adolescent and young adult populations, with some underestimation.^31–33^

### Statistical Analysis

The main exposure was the obesity phenotype variable that was empirically derived in GUTS by LCA conducted in Mplus.^24^ Fit indices (e.g., bootstrapped likelihood ratio test,^34^ entropy), class sizes, and interpretability were used to determine the appropriate number of classes. We previously reported these fit indices and the probabilities of each phenotype.^24^ Although the phenotyping analyses were initially conducted in two different age groups (9–13 years old and 14–19 years old), given the same obesity phenotypes were observed across the two age groups, the age groups were combined in the present study and the analyses were stratified by sex (as indicated by the survey participants responded to in 1996).

Associations of obesity phenotypes with incident hypertension during young adulthood (20–35 years old) were examined in SAS with logistic regression models using generalized estimating equations to account for non-independence due to sibling clusters. Participants who developed hypertension were censored at all following time points. Models subsequently adjusted for age at first follow up, duration of follow up, physical activity during childhood and adolescence, diet during childhood and adolescence, and BMI at age 18y. All analyses were stratified by sex that participants identified with in 1996.

## Results

Across both sexes, the majority of the sample was White (95%) and the mean age in 1996 was 11.6 years. During 6 years of follow-up, 10% of participants in our analytic sample developed hypertension. Females and males without overweight or obesity in childhood or adolescence had a lower risk of developing hypertension (5% and 11%, respectively) compared to those with obesity in childhood or adolescence (10% and 17%, respectively).In models predicting the development of hypertension that adjusted for age at first follow-up and duration of follow up, participants with overweight/obesity in childhood or adolescence were more likely than their peers without overweight/obesity to develop hypertension (females: odds ratio (OR) = 2.10, 95% CI (confidence interval) = 1.70, 2.58; males: OR = 1.78, 95% CI = 1.47, 2.15) (Table 2; model 1). These models were only slightly attenuated for females and males when they further adjusted for physical activity (Table 2; model 2) and diet (Table 2; model 3).

**Figure 1.**
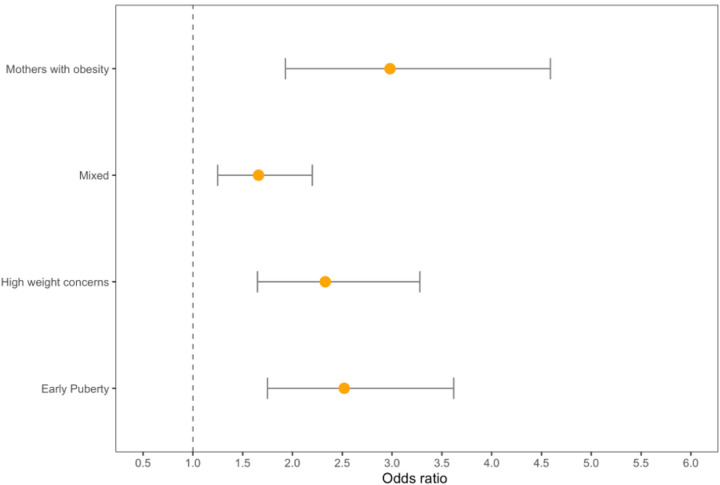
Obesity phenotypes and risk of incident hypertension in females

Among females, approximately 9–14% in each obesity subtype developed hypertension (Table 3). Female participants in all of the four empirically derived obesity phenotypes had more than twice the odds of developing hypertension in early adulthood than their peers without overweight/obesity in childhood or adolescence (early puberty subtype: OR = 2.52; 95% CI = 1.75, 3.62; mothers with obesity subtype: OR = 2.98; 95% CI = 1.93, 4.59; high weight concerns subtype: OR = 2.33; 95% CI = 1.65, 3.28; mixed subtype OR = 1.66; 95% CI = 1.25, 2.20) (Table 4; model 1; Fig. 1). Females in the mothers with obesity phenotype had a significantly greater risk of developing hypertension compared than those in the mixed subtype (*p* = 0.03).

**Figure 2.**
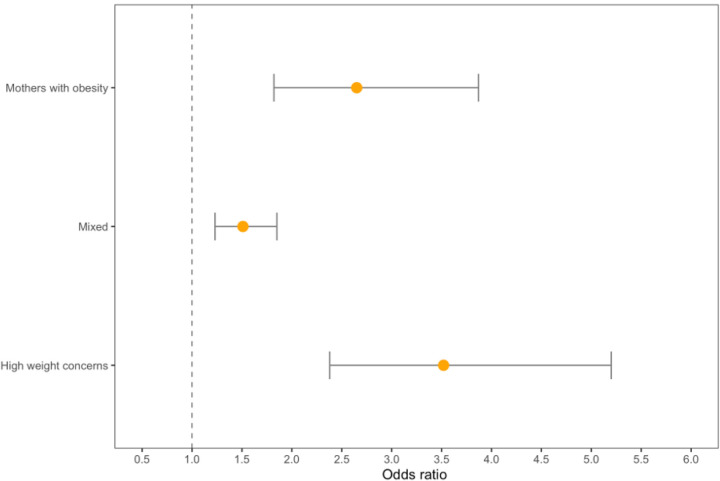
Obesity phenotypes and risk of incident hypertension in males

Among males, the mixed obesity phenotype had a significantly lower risk of hypertension incidence (15%), compared to the mothers with obesity (26%) and high weight concerns (27%) phenotypes (Table 3). Males in the mothers with obesity (OR = 2.65; 95% CI = 1.82, 3.87) and high weight concerns phenotypes (OR = 3.22; 95% CI = 2.38, 5.20) had a significantly greater risk of developing hypertension than the mixed subtype (OR = 1.51; 95% CI = 1.23, 1.86) (p = 0.004) (Table 4, model 1, Fig. 2).

Among males, but not females, the elevated risks of hypertension among those in the mothers with obesity (OR = 2.57, 95% CI = 1.38, 4.78) and high weight concerns (OR = 2.98, 95% CI = 1.53, 5.80) phenotypes remained significant even after adjusting for BMI at age 18, while the mixed subtype was attenuated and the CI crossed 1.00 (OR = 1.34, 95% CI = 0.93, 1.94), (Table 4, model 4).

## Discussion

In this prospective cohort study, females and males with overweight/obesity in childhood or adolescence were more likely than their peers without overweight/obesity to develop hypertension between ages 20 and 35. Importantly, our results show that empirically derived phenotypes of childhood and adolescent overweight/obesity had differential risks of incident hypertension in young adulthood. Among both females and males, the risk of developing hypertension was higher for those in the “early puberty”, “mothers with obesity”, and “high weight concerns” phenotypes compared to the risk of hypertension for overall overweight or obesity, indicating the importance of using empirically derived phenotypes when assessing risks of disease. Additionally, among females, the highest risks were observed among those in the “early puberty” and “mothers with obesity” phenotypes while those in the “high weight concerns” phenotype had the greatest risks of developing hypertension among males. In both sexes, participants in the “mixed” phenotypes had the lowest risks. Previous studies assessing obesity phenotypes in children have focused primarily on associations between phenotypes characterized by dietary and physical activity behaviors and weight status.^35, 36^ Our results demonstrate that there is additional heterogeneity of risk among people with obesity with other clinically discernable characteristics.

There are multiple established risk factors for hypertension in adulthood, including elevated blood pressure in childhood^37, 38^ and age at menarche.^39, 40^ Females with an early age at menarche are more likely to develop both obesity and hypertension.^41^ Some of the increase in risk of elevated blood pressure is mediated by obesity. However, the increased risk is not entirely explained by the body composition changes that accompany pubertal development in women. Even after adjusting for changes in body composition and BMI, women with an early age at menarche have an increased risk of hypertension in adulthood, compared to their peers with average or late age at menarche.^42, 43^ Another potential mechanism through which early age at menarche might act is via longer duration of exposure to high levels of estrogen, which has been found to increase the risk of developing hypertension.^44, 45^

Weight concerns are a highly prevalent source of chronic stress for many individuals in the US, yet relatively few studies have examined the associations between weight concerns and the development of hypertension.^46–49^ Most of the previous research has been among females or samples that were predominantly female. We observed that participants in the high weight concerns phenotype were more likely to develop hypertension in young adulthood compared to those without overweight/obesity in childhood or adolescence. High weight concerns, potentially due to internalized weight bias and perceived weight discrimination, can generate a chronic stress response leading to increased cortisol secretion.^50, 51^ Furthermore, previous studies have also found an obesity-independent relationship between hypertension and binge eating, which can increase oxidative and inflammatory responses.^23, 52^ Those physiological arousal processes have been linked to the development of hypertension.^53, 54^ Our findings suggest that males with obesity and weight concerns are at a substantially elevated risk of developing hypertension.

There is a strong association between parental and offspring weight status that persists into midadulthood.^55^ In addition, independent of BMI and waist circumference, parental history of hypertension is associated with increased odds of hypertension.^41, 56–58^ Because obesity is a strong risk factor for the development of hypertension, it is essential to tease apart whether the association between family history and the development of hypertension is independent of weight status. Few studies have considered both family history of hypertension and obesity. Dormanesh et al. found a family history-related elevation in risk of hypertension above and beyond the contribution of BMI.^59^ The association reflects a genetic propensity of hypertension, as well as non-genetic factors, such as shared lifestyle.^56, 60^ Our findings showing strong associations between the mothers with obesity phenotype and incident hypertension are consistent with those of Dormanesh and colleagues.

There are numerous strengths to our study, including using a large community-based sample of participants residing across all regions of the US. Second, by assessing obesity phenotypes between 9–19 years and assessing incident hypertension between 20–35 years, our study provides evidence for the temporal ordering of obesity phenotypes in childhood and adolescence and incident hypertension in young adulthood. Third, we were able to adjust our models for potential confounding variables including physical activity and diet that are often not measured in detail in other studies. Finally, given that previous studies have mostly used BMI cutoffs alone to study the association between childhood obesity and hypertension in adulthood, our study expands the literature by showing significant associations between obesity phenotypes characterized by psychological, behavioral, and familial factors and incident hypertension.

There are several limitations that warrant consideration. First, incident hypertension was self-reported, which may lead to some underestimation and misclassification of the outcome. Second, the homogeneity of our sample may limit the generalizability of our results to primarily White or economically advantaged populations. Third, some participants who were eligible for inclusion into the study were missing data on incident hypertension in adulthood and were excluded, which may result in selection bias if exclusion was related to overweight/obesity in childhood or adolescence and hypertension in young adulthood. Lastly, there is a potential for residual confounding due to other potential confounders not included in the models, including family history of hypertension.

Despite these limitations, our results offer strong support for examining the health consequences of obesity separately by obesity phenotypes rather than combining all people with obesity into a heterogenous group, which is the approach taken in most research. If these results are replicated prospectively, it may be valuable for clinicians to increase hypertension surveillance in young adults with obesity sub-phenotypes that increase risk.

## Figures and Tables

**Table 1 a.** Prevalence of indicators used to derive obesity phenotypes in childhood and adolescent among females by phenotype

Females					
	Under or normal weight in childhood and adolescence (n = 6280)	“Early Puberty” phenotype (n = 481)	“Mothers with obesity” phenotype (n = 295)	“High weight concerns” phenotype (n = 584)	“Mixed” phenotype (n = 1295)
Maternal weight status Overweight at 10 Obesity in adulthood	470 (7.5%) 723 (11.5%)	75 (15.6%) 152 (31.6%)	133 (45.1%) 280 (94.9%)	94 (16.1%) 146 (25.0%)	134 (10.4%) 231 (17.8%)
Binge eating	441 (7.0%)	25 (5.2%)	30 (10.2%)	252 (43.2%)	62 (4.8%)
High social eating influence	366 (5.8%)	57 (11.9%)	75 (25.4%)	302 (51.7%)	89 (6.9%)
High concerns with weight and shape	1553 (24.7%)	211 (43.9%)	176 (59.7%)	568 (97.3%)	436 (33.7%)
High depression	641 (10.2%)	37 (7.7%)	55 (18.6%)	256 (43.8%)	79 (6.1 %)
Pubertal Development Early	1188 (18.9%)	244 (50.7%)	58 (19.7%)	142 (24.3%)	311 (24.0%)
Age at menarche	12.5 (1.2)	10.6 (0.6)	12.0 (1.1)	12.0 (1.1)	12.4 (0.9)
Last age of follow up	32.6 (3.2)	32.3 (3.2)	32.6 (3.2)	32.2 (3.0)	32.3 (3.3)
Duration of follow up	12.0 (3.3)	11.8 (0.6)	12.0 (3.3)	11.7 (3.2)	11.8 (3.5)

**Table 1 b.** Prevalence of indicators used to derive obesity phenotypes in childhood and adolescent among males by phenotype

Males				
	Under or normal weight in childhood and adolescence (n = 4860)	“Mothers with obesity” phenotype (n = 325)	“High weight concerns” phenotype (n = 240)	“Mixed” phenotype (n = 2251)
Maternal weight status Overweight at 10 Obesity in adulthood	358 (7.4%) 559 (11.5%)	189 (58.2.%) 297 (91.4%)	55 (22.9%) 104 (43.3%)	210 (9.3%) 342 (15.2%)
Binge eating	52 (1.1%)	7 (2.2%)	49 (20.4)	51 (2.3%)
High social eating influence	71 (1.5%)	18 (5.5%)	105 (43.8%)	104 (4.6%)
High concerns with weight and shape	82 (1.7%)	38 (11.7%)	230 (95.8%)	279 (12.4%)
High depression	374 (7.7%%)	23 (7.1%)	80 (33.3%)	231 (10.3%)
Pubertal Development Early	1636 (33.7%)	154 (47.4%)	95 (39.6%)	947 (42.1%)
Last age of follow up	33.7 (3.1)	33.7 (3.2)	33.4 (3.1)	33.4 (3.3)
Duration of follow up	13.3 (3.3)	13.3 (3.4)	12.9 (3.3)	13.0 (3.5)

**Table 2:** Estimates From Logistic Regression Models Using Generalized Estimating Equations Predicting Risk of Self-Reported Hypertension between Ages 20–35*

	Females	Males
	OR (95% CI)	OR (95% CI)
Model 1	**n = 8,591**	**n = 7,842**
No overweight/obesity	1.00 (reference)	1.00 (reference)
Overweight/obesity	2.10 (1.70, 2.58)	1.78 (1.47, 2.15)
Model 2^§^	**n = 8,533**	**n = 7,752**
No overweight/obesity	1.00 (reference)	1.00 (reference)
Overweight/obesity	2.07 (1.67, 2.56)	1.78 (1.47, 2.15)
Model 3^†^	**n = 8,583**	**n = 7,824**
No overweight/obesity	1.00 (reference)	1.00 (reference)
Overweight/obesity	2.10 (1.70, 2.59)	1.78 (1.47, 2.15)

* All models controlled for age at follow up and duration of follow up

§ Model 2 further controlled for vigorous activity in childhood/adolescence

† Model 3 further controlled for diet in childhood/adolescence

**Table 3:** Incidence of reported hypertension between ages 20–35 years by obesity phenotypes

	Under or normal weight in childhood and adolescence	“Early puberty” phenotype	“Mothers with obesity” phenotype	“High weight concerns” phenotype	“Mixed” phenotype
Females	210 (5%)	40 (11 %)	28 (14%)	45 (10%)	84 (9%)
Males	245 (11 %)	—	38 (26%)	37 (27%)	176 (15%)

**Table 4:** Estimates From Logistic Regression Models Using Generalized Estimating Equations Predicting Risk of Self-Reported Hypertension between Ages 20–35 Years*

	Females	Males
	OR (95% CI)	OR (95% CI)
Model 1	**n = 6,183**	**n = 3,694**
No overweight/obesity	1.00 (reference)	1.00 (reference)
Early Puberty	2.52 (1.75, 3.62)	—
Mothers with obesity	2.98 (1.93, 4.59)	2.65 (1.82, 3.87)
High weight concerns	2.33 (1.65, 3.28)	3.52 (2.38, 5.20)
Mixed	1.66 (1.25, 2.20)	1.51 (1.23, 1.86)
Model 2^§^	**n = 6,165**	**n = 3,669**
No overweight/obesity	1.00 (reference)	1.00 (reference)
Early Puberty	2.48 (1.72, 3.57)	—
Mothers with obesity	2.92 (1.90, 4.48)	2.63 (1.80, 3.83)
High weight concerns	2.31 (1.64, 3.26)	3.51 (2.37, 5.20)
Mixed	1.64 (1.23, 2.18)	1.51 (1.22, 1.86)
Model 3^†^	**n = 6,180**	**n = 3,692**
No overweight/obesity	1.00 (reference)	1.00 (reference)
Early Puberty	2.53 (1.76, 3.64)	—
Mothers with obesity	2.96 (1.93,4.56)	2.63 (1.81, 3.83)
High weight concerns	2.35 (1.70, 3.32)	3.51 (2.38, 5.20)
Mixed	1.65 (1.25, 2.19)	1.51 (1.22, 1.86)
Model 4^‡^	**n = 3,187**	**n = 1,601**
No overweight/obesity	1.00 (reference)	1.00 (reference)
Early Puberty	1.20 (0.69, 2.10)	—
Mothers with obesity	0.64 (0.28, 1.45)	2.57 (1.38, 4.78)
High weight concerns	0.77 (0.43, 1.39)	2.98 (1.53, 5.80)
Mixed	0.79 (0.49, 1.28)	1.34 (0.93, 1.94)

* All models controlled for age at follow up and duration of follow up

§ Model 2 controlled for vigorous activity in childhood/adolescence

† Model 3 controlled for diet in childhood/adolescence

‡ Model 4 controlled for BMI at age 18

## Data Availability

The datasets generated during and/or analyzed during the current study are available in the Growing Up Today Study repository, https://gutsweb.org/
